# Cancer and physical activity: facilitating counseling

**DOI:** 10.1007/s12094-024-03795-9

**Published:** 2024-12-03

**Authors:** Alejandro Gómez-Bruton, José Manuel Granada-López, Elena Aguirre, Nuria Garatachea

**Affiliations:** 1 EXER-GENUD (Growth, Exercise, Nutrition and Development) Research Group, Faculty of Health and Sport Sciences, Department of Physiatry and Nursing, University of Zaragoza, Huesca, Spain; 2 Centro de Investigación Biomédica en Red de Fisiopatología de la Obesidad y Nutrición (CIBEROBN), Instituto de Salud Carlos III, Madrid, Spain; 3 Department of Physiatry and Nursing, University of Zaragoza, Zaragoza, Spain; 4 Research Group B53_23R: SAPIENF, Zaragoza, Spain; 5 Research Group GIISA021 Seguridad y Cuidados, Zaragoza, Spain; 6 Medical Oncology Department, Hospital Quironsalud, Zaragoza, Spain

**Keywords:** Oncology, Doctors, Physicians, Advice, Recommendations

## Abstract

Recent studies suggest that only 27.6% of cancer survivors meet PA guidelines. This could partially be attributed to the limited knowledge reported by healthcare professionals (HCP) regarding the appropriate timing, methodology, and suitability of referring cancer survivors to exercise programs or professionals. In this commentary, we aim to acknowledge the challenges that HCP may face in prescribing exercise and propose potential solutions to facilitate their efforts in this regard.

## Introduction

There are several benefits of physical activity (PA) in cancer, yet engagement levels are low. Latest global estimates show that 1.4 billion adults (27.5% of the world’s adult population) do not meet the recommended level of PA to improve and protect their health [1]. Recent studies suggest that inactivity rates in cancer survivors are also high with only 27.6% meeting PA guidelines [2]. Benefits of PA in cancer include positive effects on psychological, physical, and functional outcomes and reduces the risk of cancer recurrence and death [3]. Furthermore, only in the USA, over 46,000 annually cancer cases could be potentially avoided if the American population met the PA recommendations [4]. Despite the ample research evidence supporting mental and physical benefits of PA for cancer survivors, a systematic review on the topic reported that only 58% of health care professionals (HCP) provided PA guidance and an average of 18.3% referred patients to an exercise professional or a community-specific rehabilitation program [5]. While PA cancer guidelines exist, the translation of these guidelines to the patients has not been effective. In this short communication, we discuss PA recommendations for cancer, how HCP can support and facilitate PA and explore future research opportunities.

## The recommendation

The Clinical Oncology Society of Australia (COSA) encourages all HCP to discuss the role of PA and recommend their patients toward “at least 150 minutes of moderate intensity aerobic exercise and two or three moderate intensity resistance exercise sessions each week” [6].

More specific recommendations (including type of exercise, intensity, and duration) for the different moments of the disease have been summarized by Pollan et al. [7] during the pre-surgical period and during the adjuvant antitumor medical treatment (including chemotherapy) in cancer survivors. The dose–response of exercise and the most effective type of exercise in terms of duration and intensity are still not well-defined and will probably vary for each patient. Therefore, designing and supervising patient-specific exercise programs can be a major challenge.

Although exercise is safe, feasible, and effective for cancer patients along the course of the disease [7], a Safety Reference Guide [8] has been developed and serves as a dynamic tool for safe exercise programming in a hospital-based, medically supervised setting, particularly for high-risk patients.

The few studies that have evaluated the impact of PA counseling on PA levels have found positive outcomes in patients who received PA recommendations from their oncologists. These patients exhibited increased daily walking minutes and walking days [9], as well as a higher likelihood of modifying their exercise levels following a cancer diagnosis compared to patients who did not receive any advice [10]. Therefore, it is of critical importance that HCP recommend an increase in the PA levels of their patients. The following tools presented below aim to assist HCP in implementing this recommendation.

## Changing the message: how can we help HCP to support PA in cancer patients and survivors?

### HCP knowledge/education

A significant proportion of HCP self-report limited knowledge regarding the appropriate timing, methodology, and suitability of referring cancer survivors to exercise programs or professionals. This is evidenced by studies reporting that between 35 and 50% of HCPs lack the requisite knowledge to make informed referrals [11]. Studies assessing the preparedness of medical students to prescribe exercise showed that 34.1% of Spanish students [12] and 52% of British [13] medical students felt prepared to prescribe PA. Of the Spanish medical students (second to last year students), only 15% were able to correctly identify PA recommendations while 68% of the British students (last year students) were able to correctly identify them. Results were similarly disappointing among medical residents, with only 31% of respondents correctly identifying the levels of daily PA [14]. It is noteworthy that 94.3% of medical students and 91.0% of medical residents expressed a desire for more education content focused on PA guidelines and exercise prescription.

It is, therefore, obvious that HCP need specific formation focusing on exercise and PA guidelines. In 2019, the American College of Sports Medicine published three articles [15–17] focusing on this topic and in 2021, a group of experts summarized the evidence within these articles and interpreted the information within a rehabilitation framework to facilitate implementation within rehabilitation practice. Moreover, they gave specific recommendations for each type of cancer. This article [18] could be a good starting point for HCP that wish to give evidence-based recommendations to their patients. Another guide can be found in supplemental file II of an article developed by Fong et al. [19] aiming to facilitate PA counseling between oncology care providers and patients.

### Patient knowledge/education

HCP should try to explain the importance of changing daily habits focusing not only on increasing PA levels but also on reducing sedentary time.

Furthermore, the provision of specific free patient-oriented guides could be beneficial for patients who wish to commence an exercise program but lack the resources to engage the services of a personal trainer or specialist. Organizations and researchers should prioritize the development of exercise guidelines for different cancer types. The following examples illustrate this point.

Breast cancer guide (Spanish) from The Spanish Society of Medical Oncology (SEOM). https://seom.org/seomcms/images/stories/recursos/Guias_Nutricion_Ejercicio_Cancer_Mama.pdf

Prostate cancer guide from the University of California San Francisco. https://urology.ucsf.edu/sites/urology.ucsf.edu/files/uploaded-files/basic-page/exercise-recommendations-pamphlet.pdf

Finally, it is recommended that medical institutions endeavor to establish workshops for both HCP and patients. These workshops should provide practical examples and facilitate an improved understanding of the actions that patients can take to enhance their quality of life.

### Multidisciplinary work

Interdisciplinary teamwork (ITW) involves different experts working as a team with the purpose of recommending specific care plans and is recognized as a gold standard for the management of cancer patients [20]. Patients involved in high ITW treatments reported almost four times more positive perceptions compared to patients treated by low ITW treatments [21]. It is recommended that governments implement policies to ensure that all necessary professionals are included in the treatment of this disease. This would necessitate the presence of at least an oncologist, nurse, psychologist, physiotherapist, nutritionist and sport/exercise specialist.

Although it may appear self-evident, it is notable that in developed countries such as Spain, the role of the sport/exercise professional and nutritionist is not present in all hospitals. Consequently, the responsibility for the delivery of physical exercise and nutritional guidance falls on the shoulders of the doctor or oncologist. The integration of the aforementioned professionals is essential to guarantee recommendations based on the individual, thus achieving a personalized medicine instead of prescribing the same for all patients without taking into account their stage of the disease, characteristics, preferences, etc.

### Additional resources

Lack of HCP-patient time was reported as the most common barrier in a systematic review evaluating HCP knowledge and attitude toward PA in cancer patients [5]. The lack of time could be partially compensated for if HCP had patient-focused exercise guides or infographics to hand out to patients or if other specialists (such as sport scientists) were included in the health system.

In this line, more research is needed to develop specific resources for each type of cancer but we would recommend the following for HCP searching for general infographics to hand out:

ACSM infographic (Effects of Exercise on health-related outcomes in those with cancer). https://www.acsm.org/docs/default-source/files-for-resource-library/exercise-guidelines-cancer-infographic.pdf?sfvrsn=c48d8d86_4

Exercise is medicine infographic (Moving through cancer: Exercise for people living with and beyond cancer). https://www.exerciseismedicine.org/wp-content/uploads/2021/04/Consolidated-Infographic-for-the-ACSM-Roundtable-on-Cancer-and-Exercise.pdf

Spanish Society of Medical Oncology infographics (in Spanish) (Exercise against cancer). https://www.seom.org/ejercicio-contra-el-cancer/movimiento-seom-ejercicio-contra-el-cancer#Infografia24

## Future perspective

Despite the growing body of research investigating the benefits of exercise for cancer patients and survivors in recent years, further studies are needed to identify the optimal dose–response and establish more concrete guidelines. In addition, the most effective manner of integrating exercise into the lifestyle of patients is also a matter that requires further investigation. It is important to consider that cancer diagnosis and treatment are typically sensible moments during which patients are predisposed to change their lifestyle habits to improve their health. To achieve long-term changes in the most effective way, it is necessary to define and properly systematize personalized behavioral interventions. In this regard, randomized controlled trials to test behavioral change interventions such as PA promotion and/or sedentary behavior reduction in cancer populations in home and clinical settings are needed.

We hope this information will enable HCP to facilitate positive PA behavior change in cancer patients and survivors to enable more patients and survivors to benefit from engaging in PA.

## Data Availability

Not applicable.
